# A prediction model for sulcus-to-sulcus diameter in myopic eyes: a 1466-sample retrospective study

**DOI:** 10.1186/s12886-022-02535-3

**Published:** 2022-07-16

**Authors:** Qiu-Jian Zhu, Wei-Jian Zhu, Wen-Jing Chen, Lie Ma, You Yuan

**Affiliations:** Department of Ophthalmology, Lixiang Eye Hospital of Soochow University, 215021 Jiangsu Suzhou, China

**Keywords:** WTW, STS, Prediction model, Bland–Altman test

## Abstract

**Background:**

To establish and verify the accuracy and reliability of a sulcus-to-sulcus diameter (STS) prediction model.

**Methods:**

In this retrospective study, the prediction formula was established with the data from 1466 eyes from 733 subjects from July 2020 to April 2021 and verified with the data from 278 eyes from 139 subjects between May 2021 and June 2021. Each subject was measured with a Pentacam, IOLMaster 700, OPD-Scan III, and ultrasound biomicroscope. The prediction formulas were established with multiple linear regression, and intergroup correlation coefficients (ICCs) and Bland–Altman tests were used to assess the agreement between the predicted and actual STS (actual STS was measured by UBM).

**Results:**

The explanatory variables relevant to the horizontal STS (STSH) were the Pentacam white-to-white diameter (WTWP; standardized partial regression coefficient [β] = 0.330; *p* < 0.001), the flat K value (β = -0.211; *p* < 0.001), and the anterior corneal diameter (ACD) (β = 0.178; *p* < 0.001). The corresponding multiple regression equation was : STSH (mm) = 8.061 + 0.510 × WTWP − 0.090 × Flat K value + 0.430 × ACD. The explanatory variables relevant to the vertical STS (STSV) were the WTWP (β = 0.435; *p* < 0.001), the steep K value (β = -0.271; *p* < 0.001), and the ACD (β = 0.187; *p* < 0.001). The corresponding multiple regression equation was : STSV (mm) = 8.540 + 0.492 × WTWP − 0.075 × Steep K value + 0.329 × ACD. The bias of the predicted to the actual STSH was − 0.021, with 95% limits of agreement (95% LoA) from − 0.499 to 0.457. The bias of the predicted to the actual STSV was 0.057, with 95% LoA from − 0.462 to 0.575. The ICC was 0.883 between the predicted and actual STSH and 0.859 between the predicted and actual STSV.

**Conclusions:**

The Pentacam-measured WTW, the K value and the ACD are important for predicting the STS diameter. The prediction model has good accuracy and reliability.

**Trial registration:**

Not applicable.

## Background

By 2050, 4758 million people worldwide will be expected to develop myopia and 938 million people will have high myopia [1]. The implantable collamer lens (ICL; Staar Surgical, Monrovia, California, USA) is a safe and effective option for correcting myopia [2–5]; with no corneal excision and few high-order aberrations, it is often the first choice for surgically correcting high myopia [6, 7]. The vault refers to the distance from the posterior surface of the intraocular lens to the anterior surface of the crystalline lens and is an important indicator for evaluating safety after ICL implantation [8]. Many postoperative complications are associated with vault anomalies; a high vault can cause acute angle-closure glaucoma, pigment spread syndrome and iris atrophy, while a low vault can cause subcapsular cataract [9–14].

The improper selection of ICL size is the main cause of postoperative vault abnormalities [15]. Conventionally, the ICL size is chosen based on the white-to-white diameter (WTW) and anterior chamber depth (ACD), which is also recommended by the STAAR company. However, the accuracy of this strategy is not entirely satisfactory [16]. Nakamura et al. [17] found that only 69% of patients achieved an ideal vault using STAAR’s recommended approach. Since the haptics of the ICL are located in the ciliary sulcus, the sulcus-to-sulcus diameter (STS) is used to choose the ICL size that produces the better effect [18]. Kojima et al. [19] chose the ICL size based on the STS diameter, and subsequently, 88.9% of the implant recipients had a vault measuring between 0.15 and 1 mm. However, measurement of the STS requires the use of ultrasound biomicroscopy, an invasive test that requires very high operational skill [16].

Therefore, in this study, conventional noninvasive examination results were used to establish a prediction formula for the STS, whose accuracy was then further verified in the hope of providing additional references for surgeons for selecting the size of the ICL.

## Methods

### Study population

This retrospective study was conducted in Lixiang Eye Hospital of Soochow University, Suzhou, China. The study was approved by the Ethics Committee of Lixiang Eye Hospital of Soochow University and adhered to the tenets of the Declaration of Helsinki.

All subjects were examined preoperatively for ICL implantation; 1466 eyes from 733 subjects from July 2020 to April 2021 were recruited for the establishment of a prediction formula, and 278 eyes from 139 subjects between May 2021 and June 2021 were further selected for verification of the prediction formula. No further inclusion or exclusion criteria were applied to the study cohort.

### Measurements

All subjects underwent a complete preoperative examination, which included standard comprehensive optometry, slit-lamp microscopy, and tonometry (noncontact tonometer; NT-530, Nidek Co., Ltd., Aichi, Japan). The spherical equivalent (SE) was calculated as the original spherical power plus half of the cylindrical power. A Scheimpflug camera (Pentacam, Oculus, Germany) was used to measure the flat K, steep K, mean K, and ACD values. The crystalline lens thickness (LT) and axial length (AL) were measured using a swept-source optical coherence tomography–based biometer (IOLMaster 700, Carl Zeiss Meditec AG, Jena, Germany). The horizontal WTW distance measurements were performed with three devices: the Pentacam (WTWP), the IOLMaster 700 (WTWI) and an OPD-Scan III (Nidek Technologies, Gamagori, Japan) (WTWO). The STS was obtained by an ultrasound biomicroscope (UBM; SW-3200 L; SUOER, Tianjin, China) equipped with a 50-MHz transducer. We measured the STS horizontally and vertically (STSH and STSV) for each eye. Each examination was performed by the same experienced technician or physician.

### Establishment of a prediction formula for the STSH and STSV

Multiple linear regression was used to analyse the relationship between other factors and the STS values (STSH and STSV) and to establish the corresponding prediction formulas. The stepwise method was used to select relevant independent variables and exclude confounding parameters, with input criteria less than 0.025 and output criteria greater than 0.1.

### Assessment of the prediction formula

A total of 278 eyes was used to validate the prediction formula. The intergroup correlation coefficient (ICC) and Bland–Altman test were used to assess the agreement between the predicted and actual STS values. The cumulative percentages of eyes that had a prediction error from the targeted STS values were calculated.

### Statistical analysis

SPSS 19.0 (IBM Corp., New York, NY, USA) was used to perform the data analysis, and the Kolmogorov–Smirnov test was performed for all measurement data. Normally distributed data are expressed as the means ± standard deviation (SD); nonnormally distributed data are expressed as medians and quartiles. Pearson’s correlation test was used to analyse the relationship between the STS diameters and other ocular parameters. All tests were 2-tailed, and P values < 0.05 were considered statistically significant.

## Results

There were 1466 eyes in the model establishment group and 278 eyes in the model validation group. The baseline data of the two groups in this study are summarized in Table 1.

**Table 1:** Baseline characteristics of the study participants, Mean ± SD (Range)

Characteristics	1466 eyes for establishment	278 eyes for validation	P values
Age, years	27.82 ± 6.55 (17 to 49)	27.50 to 6.93 (17 to 49)	0.459
Sex (male/female)	507/959	96/182	0.987
Refractive error (D)			
Spherical	-7.35 ± 3.01 (-24.50 to 2.50)	-7.46 ± 2.43 (-16.00 to 1.75)	0.573
Cylindrical	-1.14 ± 0.96 (-7 to 0)	-1.34 ± 1.08 (-6.5 to 0)	0.002
Spherical equivalent	-7.92 ± 3.01 (-25.75 to 1.25)	-8.13 ± 2.46 (-17 to -1.5)	0.281
Keratometric value (D)			
Flat K	42.91 ± 1.39 (36.9 to 50.0)	42.97 ± 1.53 (39.5 to 46.7)	0.475
Steep K	44.33 ± 1.55 (37.8 to 54.4)	44.58 ± 1.73 (40.6 to 49.9)	0.014
Mean K	43.62 ± 1.42 (37.35 to 52.2)	43.78 ± 1.58 (40.1 to 48.3)	0.090
STS (mm)			
Vertical	11.96 ± 0.43 (10.61 to 13.65)	11.92 ± 0.43 (10.51 to 13.01)	0.176
Horizontal	11.49 ± 0.59 (10.14 to 12.98)	11.53 ± 0.41 (10.23 to 12.66)	0.292
IOP (mmHg)	13.30 ± 2.59 (6.0 to 22.3)	13.46 ± 2.85 (9.0 to 20.3)	0.325
AL (mm)	26.76 ± 1.56 (22.56 to 34.30)	26.70 ± 1.35 (23.52 to 31.32)	0.598
ACD (mm)	3.21 ± 0.24 (2.44 to 3.90)	3.22 ± 0.25 (2.72 to 4.03)	0.617
WTW (mm)			
Pentacam	11.59 ± 0.38 (10.4 to 12.9)	11.62 ± 0.36 (10.5 to 12.7)	0.129
OPD-Scan III	11.81 ± 0.74 (10.58 to 13.19)	11.88 ± 0.41 (10.60 to 13.13)	0.163
IOLMaster 700	11.99 ± 0.39 (10.8 to 14.7)	12.04 ± 0.38 (10.9 to 13.00)	0.066
Crystalline LT (mm)	3.70 ± 0.25 (3.09 to 4.87)	3.67 ± 0.27 (3.04 to 4.45)	0.195

*STS*  sulcus-to-sulcus diameter, *WTW* white-to-white diameter, *IOP* intraocular pressure, *AL* axial length, *ACD* anterior chamber depth, *LT* lens thickness

Table 2 shows the Pearson correlation analysis results. The STSH was correlated with age, astigmatism, the flat K, steep K and mean K values, AL, ACD, crystalline LT and three WTW values, while the STSV was correlated with age, the flat K, steep K and mean K values, IOP, AL, ACD, crystalline LT and three WTW values.

**Table 2:** Pearson correction analyse between the STSH and STSV and other parameters

	STSH (mm)		STSV (mm)	
	r	P	r	P
Age, years	-0.067	0.011	-0.126	< 0.001
Refractive errors (D)				
Spherical	0.016	0.549	0.027	0.306
Cylindrical	-0.069	0.008	0.029	0.264
Spherical equivalent	0.001	0.963	0.028	0.280
Keratometric value (D)				
Flat K	-0.379	< 0.001	-0.452	< 0.001
Steep K	-0.314	< 0.001	-0.444	< 0.001
Mean K	-0.358	< 0.001	-0.465	< 0.001
IOP (mmHg)	-0.043	0.102	-0.089	0.001
AL (mm)	0.231	< 0.001	0.273	< 0.001
ACD (mm)	0.359	< 0.001	0.418	< 0.001
WTW (mm)				
Pentacam	0.523	< 0.001	0.633	< 0.001
OPD-Scan III	0.291	< 0.001	0.355	< 0.001
IOLMaster 700	0.492	< 0.001	0.578	< 0.001
Crystalline LT (mm)	-0.070	0.008	-0.053	0.043

*STS *sulcus-to-sulcus diameter, *WTW *white-to-white diameter, *IOP*intraocular pressure, *AL*axial length, *ACD* anterior chamber depth, *LT*lens thickness

Table 3 shows the results of the stepwise multivariate regression analysis. The explanatory variables relevant to the STSH were the Pentacam WTW (WTWP) (standardized partial regression coefficient [β] = 0.330; *p* < 0.001), the flat K value (β = -0.211; *p* < 0.001), and ACD (β = 0.178; *p* < 0.001). The multiple regression equation for the STSH was expressed as follows: STSH (mm) = 8.061 + 0.510 × WTWP − 0.090 × Flat K value + 0.430 × ACD. The R, R^2^ and adjusted R^2^ values of the model were 0.563, 0.317 and 0.316, respectively. The explanatory variables relevant to the STSV were the WTWP (standardized partial regression coefficient [β] = 0.435; *p* < 0.001), the steep K value (β = -0.271; *p* < 0.001), and the ACD (β = 0.187; *p* < 0.001). The multiple regression equation for the STSV was expressed as follows: STSV (mm) = 8.540 + 0.492 × WTWP − 0.075 × Steep K value + 0.329 × ACD. The R, R^2^ and adjusted R^2^ values of the model were 0.688, 0.474 and 0.473, respectively.

**Table 3:** Stepwise multivariate regression analysis of STSH and STSV

	STSH (mm)			STSV (mm)		
	(constant = 8.061; R = 0.563; R^2^ = 0.317; adjusted R^2^ = 0.316)			(constant = 8.540; R = 0.688; R^2^ = 0.474; adjusted R^2^ = 0.473)		
	Partial regression coefficient (B)	Standardized partial regression coefficient (β)	P value	Partial regression coefficient (B)	Standardized partial regression coefficient (β)	P value
Age, years						
Refractive errors (D)						
Spherical						
Cylindrical						
Spherical equivalent						
Keratometric value (D)						
Flat K	-0.090	-0.211	< 0.001			
Steep K				-0.075	-0.271	< 0.001
Mean K						
IOP (mmHg)						
AL (mm)						
ACD (mm)	0.430	0.178	< 0.001	0.329	0.187	< 0.001
WTW (mm)						
Pentacam	0.510	0.330	< 0.001	0.492	0.435	< 0.001
OPD-Scan III						
IOLMaster 700						
Crystalline LT (mm)						

*STS*  sulcus-to-sulcus diameter, *WTW *white-to-white diameter, *IOP* intraocular pressure, *AL *axial length, *ACD*  anterior chamber depth, *LT *lens thickness

**Fig. 1:**
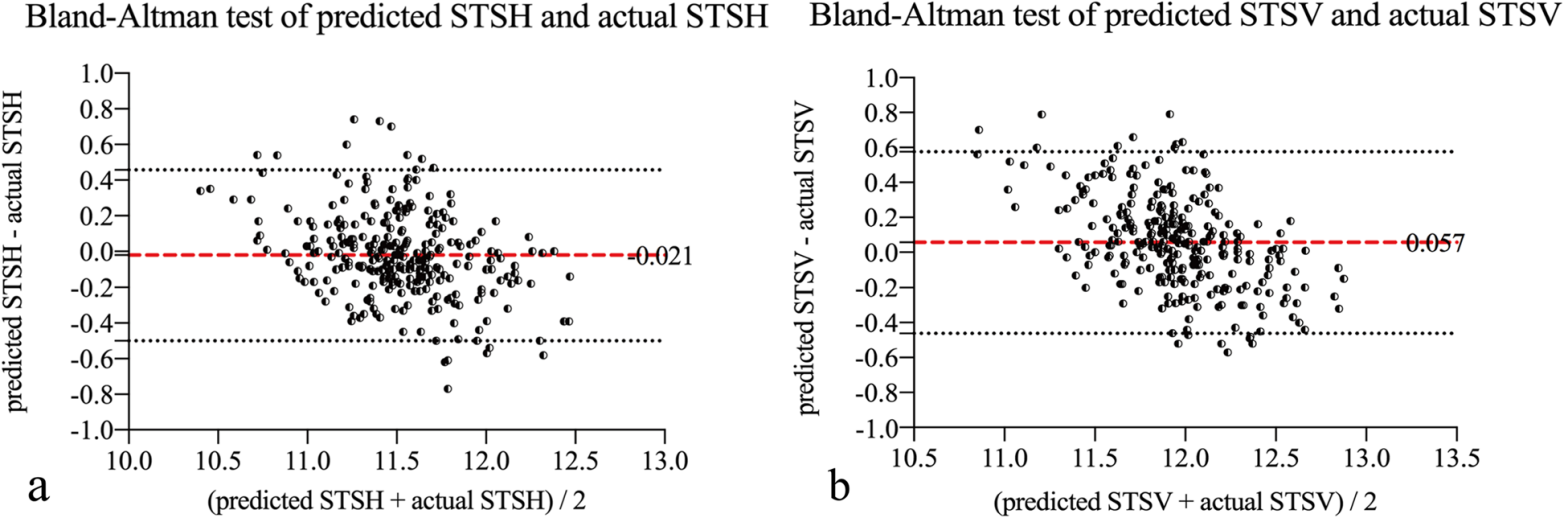
Bland–Altman test for the predicted and actual STS values. (a) shows the agreement between the predicted and actual STSH, and (b) shows the agreement between the predicted and actual STSV

Figure 1 shows the Bland–Altman plot of the predicted and actual STS values. The bias of the predicted versus the actual STSH was − 0.021, with 95% limits of agreement (95% LoA) from − 0.499 to 0.457, and the standard deviation (SD) of the bias was 0.244. The bias of the predicted versus the actual STSV was 0.057, with 95% LoA from − 0.462 to 0.575, and the SD of the bias was 0.265

**Fig. 2:**
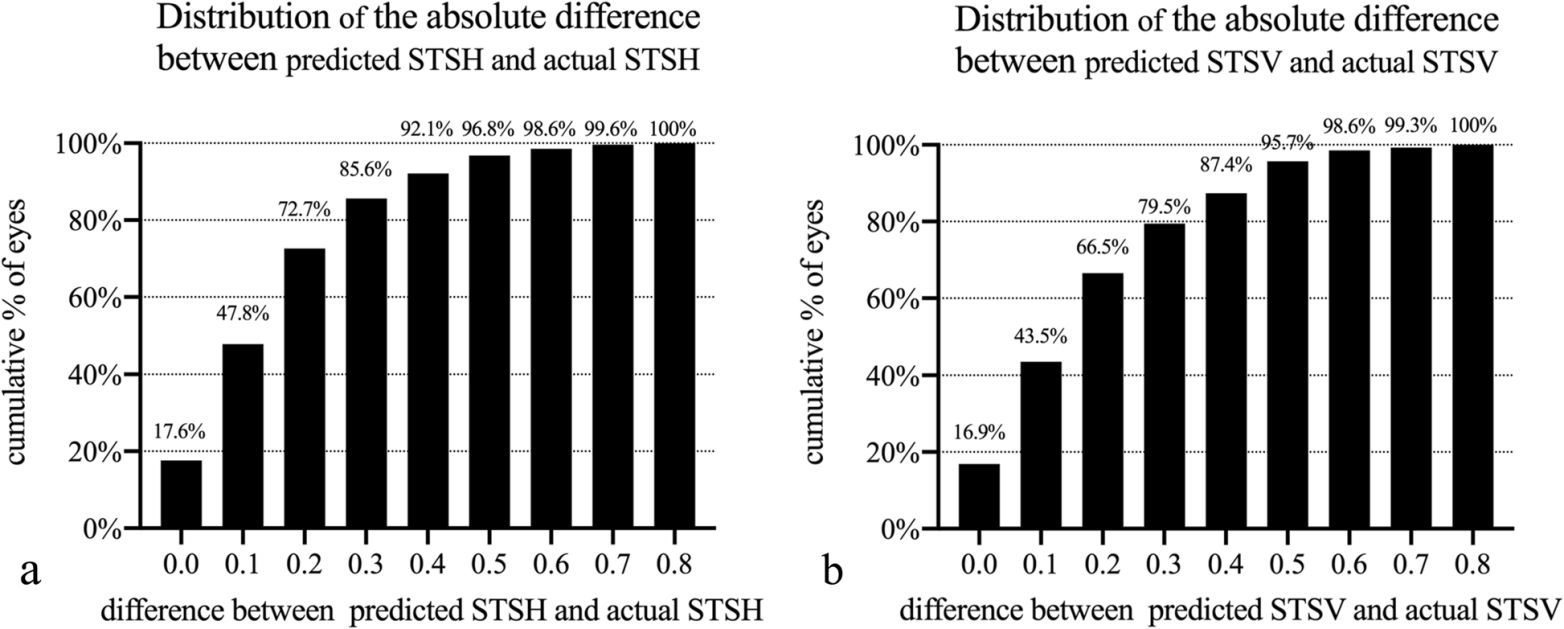
Cumulative probability distribution of the prediction error. (a) shows the cumulative probability distribution of the error between the predicted and actual STSH, and (b) shows the cumulative probability distribution of the error between the predicted and actual STSV

Figure 2 shows the cumulative probability distribution of the prediction error. For the STSH, 72.7% of eyes were within 0.2 mm, and 92.1% of eyes were within 0.4 mm. For the STSV, 66.5% of eyes were within 0.2 mm, and 87.4% of eyes were within 0.4 mm

The ICC between the predicted and actual STSH was 0.883 (95% CI: 0.852, 0.907), while that between the predicted and actual STSV was 0.859 (95% CI: 0.821, 0.888)

## Discussion

Since the ICL is implanted into the ciliary sulcus, measurement of STS is very important for predicting the subsequent vault. To date, UBMs remain the only device that can directly detect the morphology of the ciliary sulcus and measure the STS. However, the required measurements are time consuming and require considerable skill and experience, and the test is invasive, causing considerable discomfort to the patient. Therefore, our study established a prediction formula for the STS by retrospectively analysing certain noninvasive test results for a large sample size. The accuracy and reliability of the prediction formula were verified in a subsequent study. The large sample size improved the validity of the statistical analysis and made this study highly reliable.

According to previous studies, the human ciliary sulcus is vertically elliptical, and the vertical STS tends to be larger than the horizontal STS [20, 21]. According to our previous study, the vertical STS affects the vault after ICL implantation independent of the horizontal STS [22]. Therefore, in this study, separate prediction formulas were established for the horizontal and vertical STS.

According to the results of correlation and multivariate analyses, the WTWP, flat K value and ACD were the influencing factors of the STSH, producing the following regression formula: STSH (mm) = 8.061 + 0.510 × WTWP − 0.090 × flat K value + 0.430 × ACD. Furthermore, the WTWP, steep K value and ACD were the influencing factors of the STSV, producing the following regression formula: STSV (mm) = 8.540 + 0.492 × WTWP − 0.075 × Steep K value + 0.329 × ACD. This is very interesting. The most suitable instrument for measuring the WTW for selecting the size of the ICL has been a consistent point of argument in the literature, as different instruments produce significantly different WTW measurements, which thus are not completely interchangeable [23–25]. In this study, three pieces of equipment with different principles for conducting anterior segmental analysis used to measure the WTW. The Pentacam is a Scheimpflug camera that rotates around the optical axis of the eye to create a three-dimensional model of the anterior segment. The WTW is automatically measured from photographs of the anterior surface of the eye with a resolution of 0.1 mm [25]. The IOLMaster 700 is an SS-OCT-based biometer, and the limbus is used for WTW measurement via automatic detection by a digital greyscale photograph of the anterior eye segment [26]. The OPD Scan III is capable of automatically detecting the limbus by comparing greyscale steps of slit-scanning images and calculates the horizontal corneal diameter [27]. According to the results of this study, the WTWP had the highest correlation with the STS in both the horizontal and vertical directions (Pearson’s correlation coefficient 0.532 vs. 0.492, 0.291 and 0.633 vs. 0.578, 0.355, respectively). Moreover, only the WTWP entered the final results of the stepwise multiple linear regression. Therefore, we believe that compared with the other two WTW measurements, the WTWP can better predict the STS and is more suitable for ICL size selection. Furthermore, the WTWP was the most influential factor for both the STSH and STSV (standardized partial regression coefficient [β] = 0.330; *p* < 0.001 and β] = 0.435; *p *< 0.001). The WTW and STS both describe the size of the anterior segment, so the correlation between the two is unsurprising. However, most scholars believe that there is obvious bias between the WTW and STS. In Guber et al.‘s [28] study, the horizontal WTW measures obtained using the Pentacam device were significantly larger than the STS measures (bias = 0.91 mm, *P* < 0.01). Chen et al. [29] reported that the mean difference between the STS and WTW was − 0.02 +/- 0.33 (-1.36 to 1.11) mm. Hashemian et al. [30] also suggested that there was a correlation between the WTW and STS but found a significant difference in their measurements that could diminish after adjustment.

The K value is the second influencing factor of the STS. Ghoreishi et al. [31] obtained a similar result in their study and established their own predicted model: STS = 9.549 + 0.518 WTW − 0.083 mean K. However, the sample size of their study was small (58 eyes), and there was no follow-up verification. In this study, the flat K values were used to predict the horizontal STS, and the steep K values were used to predict the vertical STS. We hypothesized that this might be because most of the subjects in this study were relatively young, and astigmatism with the rule was common, while a flat K value often represents a horizontal corneal state, and a steep K value represents the corneal status in the vertical direction.

In this study, the ACD was positively correlated with and was an important parameter for predicting both the horizontal and vertical sulcus-to-sulcus diameters. A study by Kawamorita et al. [32] showed that the ACD and STS had extremely high agreement, with an intergroup correlation coefficient of 0.918. Additionally, Gao et al. [33] found that the ACD was very important for describing the difference between the WTW and STS. In their research, the WTW and ciliary sulcus diameter were 11.46 ± 0.38 and 11.57 ± 0.32 mm in the shallow anterior chamber group, 11.58 ± 0.31 and 11.77 ± 0.26 mm in the medium anterior chamber group, and 11.68 ± 0.22 and 11.91 ± 0.23 mm in the deep anterior chamber group, respectively, and they concluded that the difference between the two diameters increased with greater anterior chamber depth. In addition, another of their studies showed similar results [34]. Chen et al. [29] also suggested that as the anterior chamber depth increased, the difference between the STS and WTW increased. These results are consistent with our findings.

The Bland–Altman test results showed that the consistency of our prediction formula was satisfactory. The bias between the predicted and actual value was 0.021 for the STSH and 0.057 for the STSV. In addition, 85.6% and 79.5% of subjects, respectively, had deviations within 0.3 mm, and 92.1% and 87.4% of subjects had deviations within 0.4 mm. Hashemian et al. [30] published an adjustment formula to improve the correlation of the WTW with the ciliary sulcus diameter. After adjustment, the SD of the STS-Caliper WTW was 0.28 mm, and the 95% LoA ranged from − 0.56 to 0.54, while the SD of the STS-Orbscan WTW was 0.31 mm, and the 95% LoA ranged from − 0.61 to 0.61. In contrast, the SD of the actual STSH-predicted STSH bias in this study was 0.24 mm, and the 95% LoA ranged from − 0.499 to 0.457. In addition, the ICCs of their study were 0.775 and 0.700, whereas ours was 0.883, which showed that our prediction model had better reliability. Moreover, the sample size of Hashemian’s study was small, and they performed validation with previous data, while ours was based on a new cohort of subjects, improving the reliability of our results.

There are certain limitations in this study. First, although this study was the largest sample-size STS prediction study to date, all the people included in this study were of Han ethnicity. Whether the conclusions of this study can be applied to other ethnic groups requires further verification. Second, examination with the UBM requires highly technical expertise and experience. All the UBM examinations in this study were completed by the same experienced technician with superb skills, and whether different operators would obtain different results requires further study. Third, the findings of this study have not been introduced into clinical application. Whether this model can be helpful in ICL size selection will be the subject of a follow-up study.

In conclusion, the WTW measured by the Pentacam, the K values and the ACD are three important parameters for predicting the STS. The prediction model has good accuracy and reliability.

## Data Availability

If someone wishes to request the data from this study, please contact You Yuan (Corresponding author).

## Abbreviations

STS: Sulcus-to-sulcus diameter
WTW: White-to-white diameter
IOP: Intraocular pressure
AL: Axial length
ACD: Anterior chamber depth
LT: Lens thickness
WTWI: White-to-white diameter measured by the IOLMaster 700
WTWO: White-to-white diameter measured by the OPD-Scan III
STSH: Horizontal sulcus-to-sulcus diameter
STSV: Vertical sulcus-to-sulcus diameter
